# Genetics of Type 2 Diabetes: Implications from Large-Scale Studies

**DOI:** 10.1007/s11892-022-01462-3

**Published:** 2022-03-19

**Authors:** Natalie DeForest, Amit R. Majithia

**Affiliations:** 1 Department of Medicine, Division of Endocrinology, University of California San Diego, La Jolla, CA USA; 2 Biomedical Sciences Graduate Program, University of California San Diego, La Jolla, CA USA

**Keywords:** Type 2 diabetes, Human genetics, GWAS, Genetic risk score, Polygenic risk score, Multi-ancestry

## Abstract

**Purpose of Review:**

Type 2 diabetes (T2D) is a multifactorial, heritable syndrome characterized by dysregulated glucose homeostasis that results from impaired insulin secretion and insulin resistance. Genetic association studies have successfully identified hundreds of T2D risk loci implicating many genes in disease pathogenesis. In this review, we provide an overview of the recent T2D genetic studies from the past 3 years with particular focus on the effects of sample size and ancestral diversity on genetic discovery as well as discuss recent work on the use and limitations of genetic risk scores (GRS) for T2D risk prediction.

**Recent Findings:**

Recent large-scale, multi-ancestry genetic studies of T2D have identified over 500 novel risk loci. The genetic variants (i.e., single nucleotide polymorphisms (SNPs)) marking these novel loci in general have smaller effect sizes than previously discovered loci. Inclusion of samples from diverse ancestral backgrounds shows a few ancestry specific loci marked by common variants, but overall, the majority of loci discovered are common across ancestries. Inclusion of common variant GRS, even with hundreds of loci, does not substantially increase T2D risk prediction over standard clinical risk factors such as age and family history.

**Summary:**

Common variant association studies of T2D have now identified over 700 T2D risk loci, half of which have been discovered in the past 3 years. These recent studies demonstrate that inclusion of ancestrally diverse samples can enhance locus discovery and improve accuracy of GRS for T2D risk prediction. GRS based on common variants, however, only minimally enhances risk prediction over standard clinical risk factors.

## Type 2 Diabetes Is a Heterogeneous but Heritable Syndrome

Type 2 diabetes (T2D) is characterized by impaired glucose metabolism arising from defects in insulin resistance and secretion [1]. In clinical practice, T2D is diagnosed by elevated blood glucose levels most commonly assessed via point measurements in the fasting state or averaged over months via glycated hemoglobin (HbA1c) tests. Clinical presentation and disease progression may vary considerably among individuals, and the prevalence of T2D varies between different ethnic groups; for example, Hispanic and Black populations have higher age-adjusted T2D prevalence compared to White and Asian groups [2, 3]. Clinical complications of T2D include microvascular complications such as retinopathy, neuropathy, and nephropathy as well as macrovascular complications such as myocardial infarction and stroke [4]. Cardiovascular disease (CVD) is the leading cause of death in people with T2D who have up to a threefold increase in CVD risk as compared to people without T2D [5].

The pathogenesis of T2D involves both environmental and genetic causes. Environmental factors including obesity, stress, and lifestyle choices such as an unhealthy, energy-dense diet, and a sedentary lifestyle have been closely associated with the development of T2D [6]. The heritability of T2D ranges from 30 to 70% [7] and family history of T2D is a significant risk factor, with an approximate two-fold relative risk for siblings [8] and a three-fold increased risk for first-degree relatives of a T2D individual [9]. A handful of robust disease genes were identified by early small-scale genetic association studies for T2D [10, 11] and related Mendelian diabetes syndromes [12]. With the advent of genotyping arrays and the systematic cataloging of common genetic variation by the International HapMap project, population-scale genome-wide association studies (GWAS) became feasible, leading to the identification of hundreds of T2D associated loci [13]. This review focuses on the T2D genetic association studies conducted over the past 3 years.

## T2D Risk Loci Marked by Common Genetic Variants Are Mostly Shared Across Ancestries

In the early 2000s, collaborative efforts spanning multiple institutions across the globe coalesced into several international consortia focused on genetic mapping of T2D along with its related traits and even complications (summarized in Table 1). Initially, consortia such as DIAGRAM [14] and MAGIC [15] aggregated participants of a single ancestry (mostly northern European), but more recently, they have included participants across a variety of ancestries [16, 17•]. As of 2018, the list of T2D associations included over 200 independent loci [18] (Table 2). Subsequent studies over the past 3 years have built upon earlier work by meta-analyzing previously collected samples with samples obtained across multiple ancestries to identify an additional 500 T2D risk loci, defined as those > 500 kb and linkage disequilibrium (LD) r^2^ < 0.05 from previously reported loci (Table 2).

**Table 1:** Overview of T2D-specific and disease agnostic large-scale consortia

Abbreviation	Name	Phenotype(s)	Sequencing type	Sample size	Ancestry	Citation	Website
DIAGRAM	DIAbetes Genetics Replication And Meta-analysis	T2D	Genotype	26,676 T2D cases and 132,532 controls	European	Scott et al. *Diabetes* 2017	https://www.diagram-consortium.org/index.html
DIAMANTE	DIAbetes Meta-ANalysis of Trans-Ethnic association studies	T2D	Genotype	European: 74,124 T2D cases and 824,006 controls	African–American, East Asian, European, Hispanic, and South Asian	Mahajan et al. *Nat. Gen* 2018; Mahajan et al. med*Rxiv* 2020	-
MAGIC	Meta-Analyses of Glucose and Insulin-related traits Consortium	Glycemic traits (fasting glucose, fasting insulin, 2 h glucose, HbA1c)	Genotype	281,416 non-diabetic individuals	East Asian, Hispanic, African American, South Asian, and sub-Saharan African	Chen et al. *Nat. Gen* 2021	https://magicinvestigators.org/
SIGMA	Slim Initiative in Genomic Medicine for the Americas	T2D, cancer, kidney disease	Genotype, exome chip	8,227 T2D cases and 12,966 controls	Hispanic	The SIGMA Type 2 Diabetes Consortium. *Nature* 2014; Mercader et. al. *Diabetes* 2017	https://kp4cd.org/sigma
AGEN	Asian Genetic Epidemiology Network	T2D, cardiovascular disease	Genotype	77,418 T2D cases and 356,122 controls	East Asian	Spracklen et al. *Nature* 2020	https://blog.nus.edu.sg/agen/
T2D-GENES	Type 2 Diabetes Genetic Exploration by Next-generation sequencing in multi-Ethnic Samples	T2D	Whole exome sequencing	20,791 T2D cases and 24,440 controls	Hispanic/Latino, European, African-American, East-Asian, and South-Asian	Flannick et al. *Nature* 2019	https://www.kp4cd.org/about/t2d
MEDIA	African Americans from the MEta-analysis of type 2 DIabetes in African Americans	T2D	Genotype	8,284 T2D cases and 15,543 controls	African American	Ng et al. *PLoS Genet* 2015	-
MVP	Million Veteran Program	Disease agnostic	Genotype	825,000	Predominantly European, also African American, Hispanic, and Asian	Gaziano et al. *J Clin Epidemiol* 2016	https://www.research.va.gov/mvp/
UKB	UK Biobank	Disease agnostic	Genotype, whole exome sequencing, whole genome sequencing	Genotype: 500,000; Whole exome: 450,000; Whole genome: 150,000	Predominantly European, also African American, Hispanic, Asian, African, and Carribbean	Bycroft et al. *Nature* 2018	https://www.ukbiobank.ac.uk/
BBJ	BioBank Japan	Disease agnostic	Genotype, whole genome sequencing	Genotype: 200,000; Whole genome: 2,000	Japanese	Nagai et al. *J Epidemiol* 2017	http://jenger.riken.jp/en/
PAGE	Population Architecture Genomics and Epidemiology	Disease agnostic	Genotype	50,000	European, African American, Asian, Native Hawaiian, and Hispanic	Wojcik et al. *Nature* 2019	https://www.pagestudy.org/

**Table 2:** Summary of recent large-scale T2D genetic association studies

Citation	Cohort(s)	Meta-analysis sample size	Ancestry	Phenotype(s)	Total risk loci	Novel risk loci
Mahajan et al. *Nat. Gen* 2018	DIAGRAM	74,124 T2D cases and 824,006 controls	European	T2D	243	135
Suzuki et al. *Nat. Gen* 2019	BBJ, Osaka-Midousuji Rotary Club, Pharma SNP, ToMMo, IIMM, JPHC, and J-MICC	36,614 T2D cases and 155,150 controls	Japanese	T2D	88	28
Spracklen et al. *Nature* 2020	AGEN, DIAMANTE, CKB, KBA, and BBJ	77,418 T2D cases and 356,122 controls	East Asian	T2D	183	61
Vujkovic et al. *Nat. Gen* 2020	MVP, DIAMANTE, Penn Medicine Biobank, Pakistan Genomic Resource, BBJ, Malmö Diet and Cancer Study, Medstar, and PennCath	228,499 T2D cases and 1,178,783 controls	European, African American, Hispanic, South Asian, and East Asian	T2D	568	318
Polfus et al. *HGG Advances* 2021	PAGE, DIAGRAM; Replication: 23andMe, DIAMANTE, SIGMA, AGEN, MEDIA	53,102 T2D cases and 193,679 controls	European, African American, Hispanic, Asian, and Native Hawaiian	T2D	39	4; 2 of which replicated
Chen et al. *Nat. Gen* 2021	MAGIC	281,416 non-T2D individuals	European, African American, sub-Saharan African, Hispanic, South Asian, and East Asian	Fasting glucose, fasting insulin, 2-h glucose, HbA1c	242	72 (99 at time of writing)

Abbreviations: *BBJ*, Biobank Japan; *ToMMo*, Tohoku Medical Megabank Organization; *IMM*, Iwate Tohoku Medical Megabank Organization; *JPHC*, Japan Public Health Center–based Prospective; *J-MICC*, Japan Multi-Institutional Collaborative Cohort; *AGEN*, Asian Genetic Epidemiology Network; *DIAMANTE*, DIAbetes Meta-ANalysis of Trans-Ethnic association studies; *CKB*, China Kadoorie Biobank; *KBA*, Korea Biobank Array; *SIGMA*, Slim Initiative in Genomic Medicine for the Americas; *MEDIA*, African Americans from the MEta-analysis of type 2 DIabetes in African Americans

The largest T2D genetic association study to date meta-analyzed GWAS from eight cohorts including population-based biobanks such as the Million Veteran Program (MVP) and Biobank Japan as well as dedicated T2D case–control cohorts such as DIAMANTE [19•]. These cohorts contained individuals from five different ancestral groups (European, African American, Hispanic, South Asian, and East Asian) for a total of 228,499 T2D cases and 1,178,783 controls. A total of 568 T2D risk loci were identified at genome-wide significance, 293 of which were novel in this study [19•]. These newly identified loci had smaller effect sizes (average beta regression coefficient of 0.032 ± 0.012 per allele) than previously discovered T2D risk loci (average beta of 0.054 ± 0.045 per allele), demonstrating that increased sample size enhanced statistical power to detect association signals with smaller biological effects. Additionally, within the MVP cohort, Vujkovic et al. performed ancestry-specific GWAS which identified an additional 21 loci in Europeans and 4 loci in African Americans not initially identified in the original meta-analysis. A few loci demonstrated higher effect sizes for T2D in African Americans compared with Europeans, but the majority of loci (92.1%) showed no significant heterogeneity in effect estimates between Europeans and African Americans.

The most recently published multi-ancestry T2D case–control genetic study illustrates the dominant effect of sample size in driving locus discovery [20]. Polfus et al. conducted a GWAS meta-analysis of 53,102 T2D cases and 193,679 controls from the multi-ethnic Population Architecture Genomics and Epidemiology (PAGE) consortium along with the DIAGRAM consortium, and replicated their findings in independent ancestry-specific samples from multiple T2D consortia including DIAMANTE, Asian Genetic Epidemiology Network (AGEN), Slim Initiative in Genomic Medicine for the Americas (SIGMA), and African Americans from the MEta-analysis of type 2 DIabetes in African Americans (MEDIA) [20]. They identified four novel loci from the discovery PAGE + DIAGRAM GWAS, two of which replicated in single ancestry replication GWAS: (1) rs11466334 near the transforming growth factor beta-1 (*TGFB1*) gene and (2) rs13052926 near beta-secretase 2 (*BACE2*). Only the *TGFB1* locus (rs11466334) was an ancestry-specific variant occurring more commonly in African (minor allele frequency (MAF) = 6.8%) and Hispanic populations (MAF = 1.3%) as compared with other ancestries (MAF < 1%). The single nucleotide polymorphism (SNP) was also predicted to be functionally consequential via disrupting a CCCTC-binding factor (CTCF) binding motif potentially leading to altered enhancer-promoter interactions. Although this study identified four novel loci, it did not re-identify over 90% of the genome-wide significant loci identified in previous studies [18, 19•] (Table 2). The critical distinguishing factor was the sample size, highlighting this as the major determinant of genetic discovery in common variant association studies for T2D.

To examine the effect of ancestry on loci associated with glycemic traits (fasting glucose, fasting insulin, 2-h glucose, and HbA1c) in non-diabetic individuals, Chen et al. and the MAGIC investigators first conducted meta-analyses of GWAS within each of the following single-ancestry populations: European, African American, Hispanic, East Asian, or South Asian. They then meta-analyzed these “single-ancestry GWAS” in a “trans-ancestry” GWAS consisting of a total of 281,416 non-diabetic individuals [17•]. From the trans-ancestry GWAS, they identified 235 loci associated with at least one glycemic trait, and 7 additional loci from the single-ancestry GWAS that did not rise to genome-wide significance in the trans-ancestry analysis. Interestingly, the single-ancestry loci had similar allele frequencies across the sampled ancestries, potentially suggesting epistatic effects with other ancestry-specific variants or that they rose to significance in a particular single-ancestry analysis simply by chance.

Of the 235 trans-ancestry glycemic trait-associated loci, 93 were novel at the time of publication and Chen et al. performed an instructive simulation to quantify the benefit of including multiple ancestries as opposed to simply increasing sample size to enhance novel locus discovery. By re-scaling the standard errors of the European single-ancestry GWAS to simulate the trans-ancestry sample size, Chen et al. found that that 21 out of the 93 (22.6%) newly discovered trans-ancestry loci would not have been identified in a GWAS restricted to European ancestry. This suggests that while the majority of novel loci were identified due to increase in sample size, a modest benefit was obtained by including non-European samples.

Furthermore, this study examined the effect of single- versus trans-ancestry analyses on the resolution of genetic fine-mapping to identify causal variants. To do this, the authors identified 98 locus-trait associations that had a single causal variant from both single- and trans-ancestry fine-mapping and found that 72 (73%) locus-trait associations showed improvements in the resolution of fine-mapping, as quantified by a decreased number of variants in the 99% credible sets. Of these 72 locus-trait associations, 53% were improved due to larger sample size in the trans-ancestry analysis and 47% were improved due to the inclusion of diverse ancestries as demonstrated by a decrease in the median number of variants in the 99% credible sets from 24 to 15 variants (37.5% median reduction). Thus, for about half the loci identified, inclusion of diverse ancestries enabled a reduction of about 10 variants from the final 99% credible sets for the causal variant.

In addition to the above-described multi-ancestry studies, recent large-scale T2D genetic studies have also been performed in East Asian populations which have been previously under-represented in GWAS. In a T2D case–control GWAS meta-analysis including Biobank Japan participants, Suzuki et al. examined 36,614 T2D cases and 155,150 controls of Japanese ancestry and identified 88 T2D risk loci, 28 of which were novel [21]. The majority (77%) of the identified lead variants are common (MAF > 0.05) in both Japanese and European populations, and Suzuki et al. demonstrated that effect sizes are strongly correlated (Pearson’s *r* = 0.83, *P* = 8.7e-51) and directly consistent (94%) between the Japanese GWAS and a comparable T2D European GWAS, indicating that the majority of genetic susceptibility between Japanese and European ancestry is shared. In addition to this study in Japanese individuals, the largest meta-analysis of T2D GWAS in individuals of East Asian ancestry to date examined 77,418 T2D cases and 356,122 controls across 23 studies including AGEN and Biobank Japan to identify 183 loci, of which 61 were novel [22•]. Upon comparison with a previously published T2D GWAS in European individuals of similar sample size, Spracklen et al. demonstrated that effect sizes of variants significantly associated with T2D in both East Asian and European ancestry were strongly correlated (*r* = 0.87). Furthermore, the authors find that only 8.4% of variants showed significant heterogeneity in effect size between the East Asian and European GWAS results, and the variants which have the greatest differences in effect sizes between the two populations are those that are common or low-frequency in East Asians but rare in Europeans (MAF < 0.1%). Overall, these recent T2D genetic association studies in East Asian ancestry cohorts underscore the finding that genetic susceptibility to T2D captured by common genetic variation is mostly shared across ancestries.

## Genetic Risk Scores for T2D Do Not Substantially Enhance Risk Prediction over Traditional Clinical Risk Factors

While over 700 loci identified by common variant association studies (i.e., GWAS) combine to explain almost 20% of T2D heritability [19•], each individual common variant (i.e., SNP) has a small to modest effect (10–30%) on disease risk as compared to simply knowing family history of T2D, which if present in a parent confers a large increase in risk (~ two–threefold)[23]. Combining multiple variants genotyped in a single person into a genetic risk score (GRS, also commonly referred to as polygenic risk score) is a logical strategy to enhance the clinical utility of genetic information from common variants to identify individuals at high risk [24]. GRS combining multiple loci were initially tested in the early 2000s with the first T2D GWAS studies. One of the first studies calculated a T2D GRS from a combination of 18 loci finding that genetic information minimally enhanced risk prediction when combined with traditional clinical risk factors such as age, sex, or family history of diabetes [25]. In the past few years, there has been a resurgence of interest in GRS leveraging many more loci identified from large-scale, multi-ancestry cohorts.

In the largest T2D GWAS to date (Table 2), Vujkovic et al. used results from a previous European GWAS [18] to calculate GRS for participants in the MVP and demonstrated that individuals with the highest T2D GRS (90–100% GRS percentile) presented the highest risk for T2D (OR = 5.21, 95% CI = 4.94–5.49) compared to those with the lowest T2D GRS (0–10% GRS percentile) [19•]. Using the GWAS effect estimates from the T2D GWAS conducted by Vujkovic et al., Polfus et al. computed a GRS for T2D in a multi-ethnic cohort [20]. From this, they found that GRS constructed from multi-ethnic computed weights demonstrated nominal increases in predictive power compared to single-ancestry computed weights, and observed strongly significant heterogeneity across ancestries for accuracy of T2D risk prediction. For instance, the multi-ethnic GRS without adjustment for clinical risk factors performed best in European and East Asian populations (AUC = 0.66 and 0.63, respectively) and most poorly in African Americans (AUC = 0.57).

**Fig. 1:**
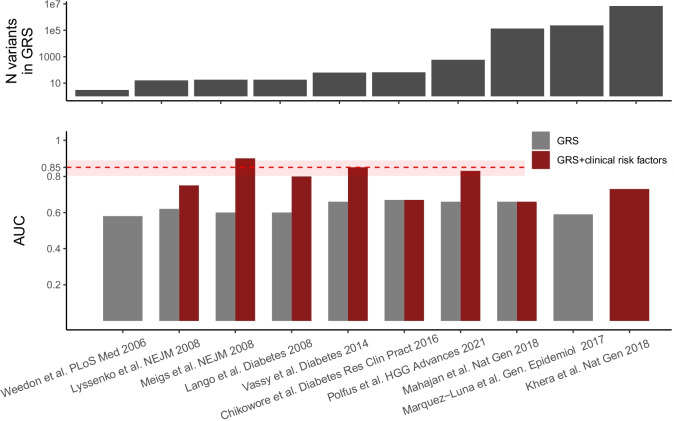
Genetic risk scores for T2D do not substantially enhance risk prediction over traditional clinical risk factors. Displayed are the outcomes from independent, large-scale studies which have constructed genetic risk scores (GRS) for T2D [18, 20, 25–27, 33–37]. Studies are shown along the x-axis, ordered by the number of variants used to construct the genetic risk scores (top panel). (bottom panel) The accuracy of the GRS alone and the GRS with clinical T2D risk factors to predict T2D from each study, quantified by the area under the receiver operating characteristic curve (AUC). The dashed red line and shaded red box represent the current predictive power and 95% confidence interval respectively of T2D clinical risk factors (age, sex, parental T2D, BMI, systolic blood pressure, fasting glucose, HDL cholesterol, and triglycerides) to predict T2D [36]

These recent studies which have generated GRS for T2D and its related phenotypes have demonstrated that GRS has the highest discriminative ability when applied to European populations and that performance is subsequently improved in non-European ancestries when GRS is computed using multi-ancestry weights [20, 26]. However, even after a decade of methodological refinement as well as an increase in the number of loci to calculate GRS, the predictive power of GRS for T2D is comparable to discrimination by clinical risk factors alone (Fig. 1). However, GRS may have a role in detecting individuals at high risk before clinical risk factors become apparent. Whether information from GRS can motivate preventative therapy to meaningfully reduce rates of future incident T2D remains to be studied. GRS have also been applied widely beyond T2D to other heritable diseases such as heart disease and cancer [27, 28] and even been offered as a tool for embryo screening during in vitro fertilization [29–31]. But the current consensus among geneticists, ethicists, and clinicians is that the scientific and technical uncertainty in GRS and their limited predictive power should limit their use in genetic screening [32].

## Perspective on Future Genetic Mapping Studies in T2D

With over 700 T2D risk loci identified by common variant genetic association studies (i.e., GWAS), decades of follow-up biological studies in cellular and organismal model systems will be required to fully understand the causal genes and molecular mechanisms of disease pathogenesis. Thus, it is unlikely that simply aggregating larger T2D case:control cohorts for association analysis will provide scientific and clinical insight into T2D. Here, we expect that an enhanced focus on T2D complications, which are the leading cause of death in T2D[5] and are independently heritable of diabetes[38], using common variant association methodology will advance understanding and treatment as has been ongoing for T1D [39].

It has long been appreciated that T2D is a highly heterogeneous disorder classically defined along a spectrum of insulin secretion and insulin resistance which ultimately belies differences in clinical presentation, disease progression, response to treatment, and susceptibility to complications [40]. Recent work added four clinically available variables to insulin and glucose to refine T2D subtypes which were shown to differ in patient characteristics and risk of comorbidities [41]. Genetic association analysis of these T2D subtypes has revealed partially distinct genetic backgrounds and heritability demonstrating progress in refining T2D classification to reduce clinical heterogeneity [42]. We expect that the use of omics measurements such as transcriptomics, proteomics, and metabolomics applied to blood samples will enable the identification of novel patterns to resolve T2D heterogeneity and in combination with genetic association methodologies enable identification of distinct biological pathways. Early efforts in the application of metabolic measurements to fasting and postprandial samples in concert with GWAS have shown the potential of such omics approaches [43].

In contrast to common variants which were generated millions of years ago in an genetically equilibrated ancestral human population, rare genetic variants (MAF << 0.01) which arose during the “out of Africa” human population expansion [44] potentially offer different mechanisms of disease causation. As exome and whole genome sequencing are becoming more commonplace, investigators have begun to examine rare variant associations with T2D [45]. The challenge with rare variant association studies is that the sample size requirement vastly increases due to the low allele frequency and increase in multiple hypothesis testing burden from the large number of rare variants [46]. Using a combination of methodological enhancements such as “burden tests” which aggregate rare variants across a gene to reduce the multiple hypothesis testing burden and population-scale biobanks like the UK Biobank to increase sample size, investigators have identified novel T2D loci such as *GIGYF1* [47] and *FAM234A* [48] which were not marked by common variant signals.

In summary, we expect that large-scale exome and whole-genome sequencing of population scale biobanks will facilitate rare-variant association studies of T2D to identify novel loci beyond what has been identified by common variant association studies thus far. Additionally, focusing genetic mapping efforts on micro- and macrovascular diabetes complications is likely to maximize the value of novel locus discovery to further understand and treat T2D.
